# Whole‐genome circulating tumor DNA methylation landscape reveals sensitive biomarkers of breast cancer

**DOI:** 10.1002/mco2.134

**Published:** 2022-06-17

**Authors:** Luo Hai, Lingyu Li, Zongzhi Liu, Zhongsheng Tong, Yingli Sun

**Affiliations:** ^1^ Central Laboratory, National Cancer Center/National Clinical Research Center for Cancer/Cancer Hospital and Shenzhen Hospital Chinese Academy of Medical Sciences and Peking Union Medical College Shenzhen China; ^2^ Department of Breast Oncology Tianjin Medical University Cancer Institute and Hospital Tianjin China; ^3^ University of Chinese Academy of Sciences Beijing China; ^4^ CAS Key Laboratory of Genome Sciences and Information, Beijing Institute of Genomics Chinese Academy of Sciences Beijing China

**Keywords:** biomarkers, breast cancer, circulating tumor DNA, early detection

## Abstract

The changes in circulating tumor DNA (ctDNA) methylation are believed to be early events in breast cancer initiation, which makes them suitable as promising biomarkers for early diagnosis. However, applying ctDNA in breast cancer early diagnosis remains highly challenging due to the contamination of background DNA from blood and low DNA methylation signals. Here, we report an improved way to extract ctDNA, reduce background contamination, and build a whole‐genome bisulfite sequencing (WGBS) library from different stages of breast cancer. We first compared the DNA methylation data of 74 breast cancer patients with those of seven normal controls to screen candidate methylation CpG site biomarkers for breast cancer diagnosis. The obtained 26 candidate ctDNA methylation biomarkers produced high accuracy in breast cancer patients (area under the curve [AUC] = 0.889; sensitivity: 100%; specificity: 75%). Furthermore, we revealed potential ctDNA methylated CpG sites for detecting early‐stage breast cancer (AUC = 0.783; sensitivity: 93.44%; specificity: 50%). In addition, different subtypes of breast cancer could be well distinguished by the ctDNA methylome, which was obtained through our improved ctDNA‐WGBS method. Overall, we identified high specificity and sensitivity breast cancer‐specific methylation CpG site biomarkers, and they will be expected to have the potential to be translated to clinical practice.

## Graphical abstract

This study demonstrates the utility of circulating tumor DNA (ctDNA) methylation analysis in early detection and molecular subtyping for breast cancer. The improved ctDNA whole‐genome bisulfite sequencing method was effective at developing novel sensitive and specific biomarkers for the diagnosis, early detection, and molecular subtyping of breast cancer. We anticipate that the method and diagnostic biomarkers of breast cancer have substantial clinical translation potential.

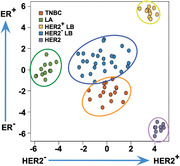

## 1 INTRODUCTION

Breast cancer is the most common cancer with the second‐highest cancer‐related morbidity in women worldwide.^1^ Early diagnosis followed by timely treatment can significantly improve the survival of breast cancer patients. To date, clinical methods for breast cancer detection include medical imaging detection (e.g., ultrasonic testing, X‐ray imaging, computed tomography, magnetic resonance imaging, positron emission tomography‐computed tomography),^2^, ^3^, ^4^, ^5^, ^6^ serum antigen protein markers (e.g., cancer antigen 15‐3, carcinoembryonic antigen, and cancer antigen 125),^7^ and tissue biopsy.^8^ These methods have their advantages but also have inevitable disadvantages. For example, medical imaging clearly shows the morphology and location of tumor tissues. However, imaging detection may cause harm to patients when using contrast agents and high‐energy rays, and medical imaging detection usually lags behind tumor progression.^9^ Serum antigen protein markers have broad applications. Nevertheless, those serum biomarkers may underevaluate tumor heterogeneity, resulting in higher misdiagnosis rates for breast cancer.^10^ At present, tissue biopsy is the “gold standard” method. However, biopsy is expensive and invasive and has an unignorable false‐negative rate in small tumors.^11^ Additionally, punctures in tumors might also lead to postoperative bleeding, pain, and infection.^12^ Meanwhile, single‐site biopsy cannot reveal intratumor and multimetastatic genetic and phenotypic heterogeneity in breast cancer processing, resulting in the loss of potential therapeutic targets and opportunities.^13^ Overall, conventional biomarkers have been applied for a long time in clinical diagnosis and have the value of continued application. Nevertheless, there is still an urgent need to explore novel biomarkers with noninvasive sampling, high specificity and sensitivity for early breast cancer diagnosis and classification.

Tumor tissue can release one‐ or two‐strand DNA fragments named circulating tumor DNA (ctDNA) into the body fluid, which offers a new test approach.^14^ There are many apparent advantages of ctDNA as a surrogate for current clinical methods: (i) Blood collection with ctDNA is noninvasive. (ii) The 2‐hour half‐life of ctDNA in plasma allows it to be used for real‐time and dynamic monitoring of tumor progression.^15^ (iii) ctDNA can reflect relapse after surgery ahead of medical imaging for several months.^16^ (iv) ctDNA detection shrinks the bias caused by intratumoral genetic heterogeneity. Because ctDNA has the above advantages, many attempts are ongoing to use ctDNA as an early diagnostic biomarker of breast cancer.^17^, ^18^, ^19^

DNA methylation has been reported to play essential roles in tumor occurrence and development.^20^, ^21^, ^22^ A previous study demonstrated that the accuracy of DNA methylation in predicting the risk of breast cancer is better than that of copy number variants by tissue.^23^ In addition to genomic variations, an increasing number of studies have reported the potential application of ctDNA methylation as a biomarker of various cancers, including breast cancer,^24^ hepatocellular cancer,^25^ lung cancer,^26^ and colon cancer.^27^ However, routine whole‐genome bisulfite sequencing (WGBS) is difficult to detect, limited by the low concentration of ctDNA in blood and background contamination.^28^ Here, we improved the ctDNA extraction method, and only fragments of approximately 160–180 bp were applied in the subsequent ctDNA WGBS library construction. We also increased the sequencing depths to obtain better signals for data analysis. With the above efforts, we could obtain the whole‐genome DNA methylation landscape of breast cancer and reveal biomarkers for breast cancer, including early‐stage breast cancer. In addition, different subtypes of breast cancer could be distinguished by the ctDNA methylome. Our work showed that the ctDNA methylome could be a promising noninvasive method to detect breast cancer.

## 2 RESULTS

### 2.1 An improved method was developed to obtain ctDNA with less background contamination and a high‐quality WGBS library for sequencing

**FIGURE 1:**
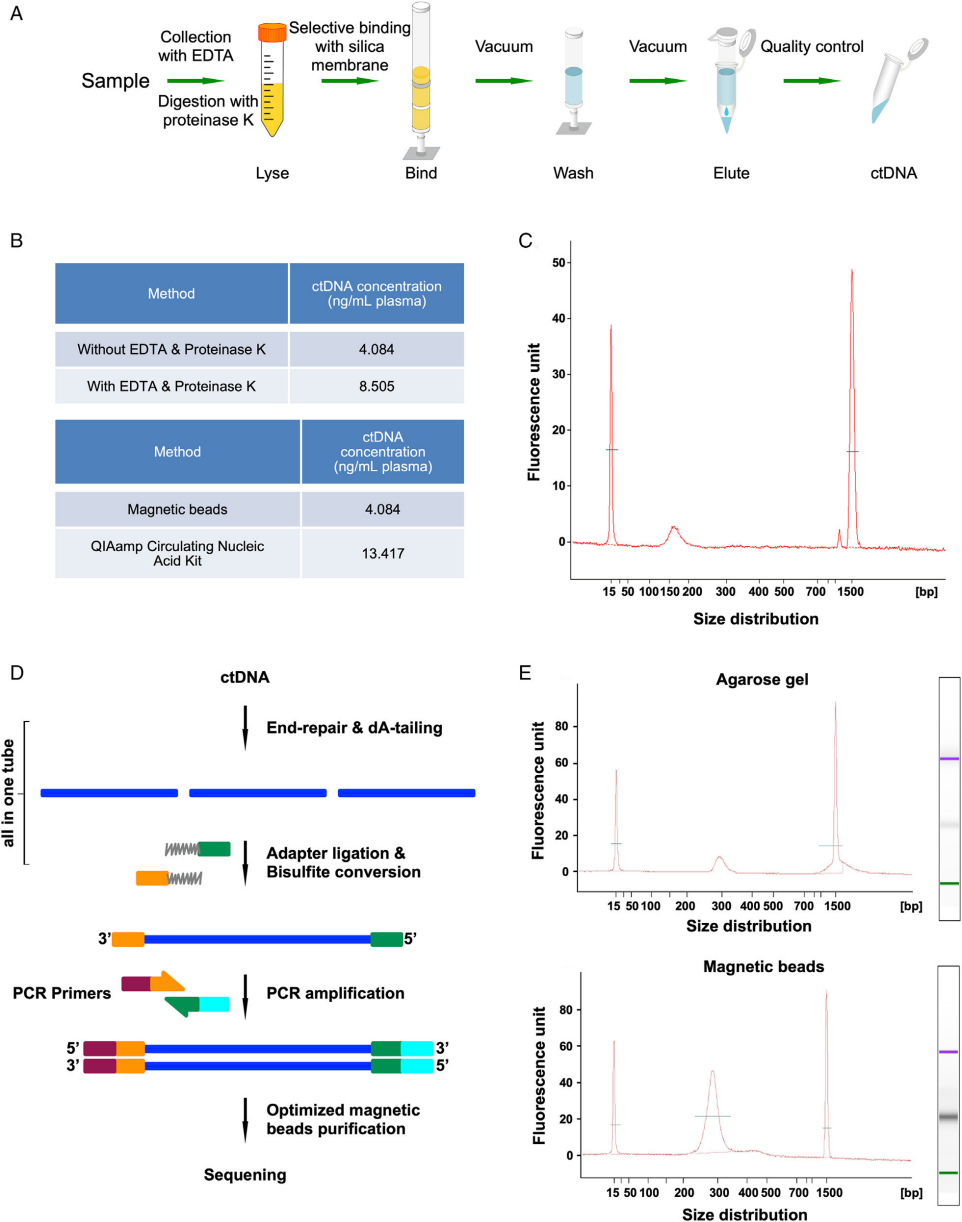
Optimization of circulating tumor DNA (ctDNA) extraction and whole‐genome bisulfite sequencing (WGBS) library preparation. (A) The workflow shows the processing of ctDNA extraction from plasma. (B) The comparison of ctDNA extraction method: with EDTA and proteinase K versus without EDTA and proteinase K; magnetic beads method versus QIAamp circulating nucleic acid kit method. (C) Extracted plasma ctDNA has sharp peaks of approximately 160–180 bp, indicating that the obtained DNA fragments are high‐quality ctDNA. (D) The workflow shows the process of ctDNA methylation library construction. (E) The head‐to‐head comparison showed that the magnetic bead method significantly increased the recovery ratio of the resulting library compared to the agarose gel method

We developed an improved method to build a WGBS library in trace quantities of ctDNA. We utilized the selective binding properties of the silica membrane to extract ctDNA from plasma using QIAamp circulating nucleic acid kits (Figure 1A). First, the plasma samples were lysed in an optimized buffer and adjusted to binding conditions. Then, the samples were loaded directly onto a spin column. In this step, ctDNA was bound to the silica membrane, and contaminants were completely removed in wash steps. Finally, pure ctDNA was eluted in small volumes of a low‐salt buffer for downstream applications. Meanwhile, we optimized the extraction method of ctDNA and found that higher amounts of ctDNA could be extracted by adding EDTA and proteinase K (Figure 1B). The explanation for the improvement in ctDNA extraction performance might be that the addition of EDTA could inhibit the degradation of ctDNA by nucleases in blood. In addition, proteinase K could improve ctDNA yield by releasing protein‐bound ctDNA. Moreover, the ctDNA amount obtained was higher using the QIAamp circulating nucleic acid kit‐based protocol than the conventional magnetic‐bead‐based protocol. The quality control analysis demonstrated that the DNA fragments have sharp peaks of approximately 160–180 bp (Figure 1C), indicating that our method could obtain high‐quality ctDNA.

As shown in Figure 1D, purified ctDNA was ligated with an adapter and converted using bisulfate with an “all‐in‐one tube” operation. Specifically, the process of end repair, dA‐tailing, adapter ligation, and bisulfite conversion was performed in the same tube, considering fewer ctDNA requirements and material losses. Then, the fragments were completed by PCR amplification, followed by purification. We optimized the purification method of the ctDNA library by comparing the agarose gel extraction method with the magnetic bead method. The head‐to‐head comparison showed that the magnetic bead method significantly increased the recovery ratio of the resulting library (Figure 1E). Bisulfite treatment of ctDNA led to the conversion of unmodified cytosines to uracil while keeping 5‐methylcytosine unchanged, which could be mapped at a single base resolution after PCR and sequencing. With the above efforts, our ctDNA‐WGBS method could be applied for whole‐genome base‐level resolution detection of 5‐methylcytosine in ctDNA from breast cancer patients. Eventually, we could obtain the whole‐genome DNA methylation landscape of breast cancer and reveal potential biomarkers.

### 2.2 ctDNA methylation biomarkers enabled sensitive detection of breast cancer

**FIGURE 2:**
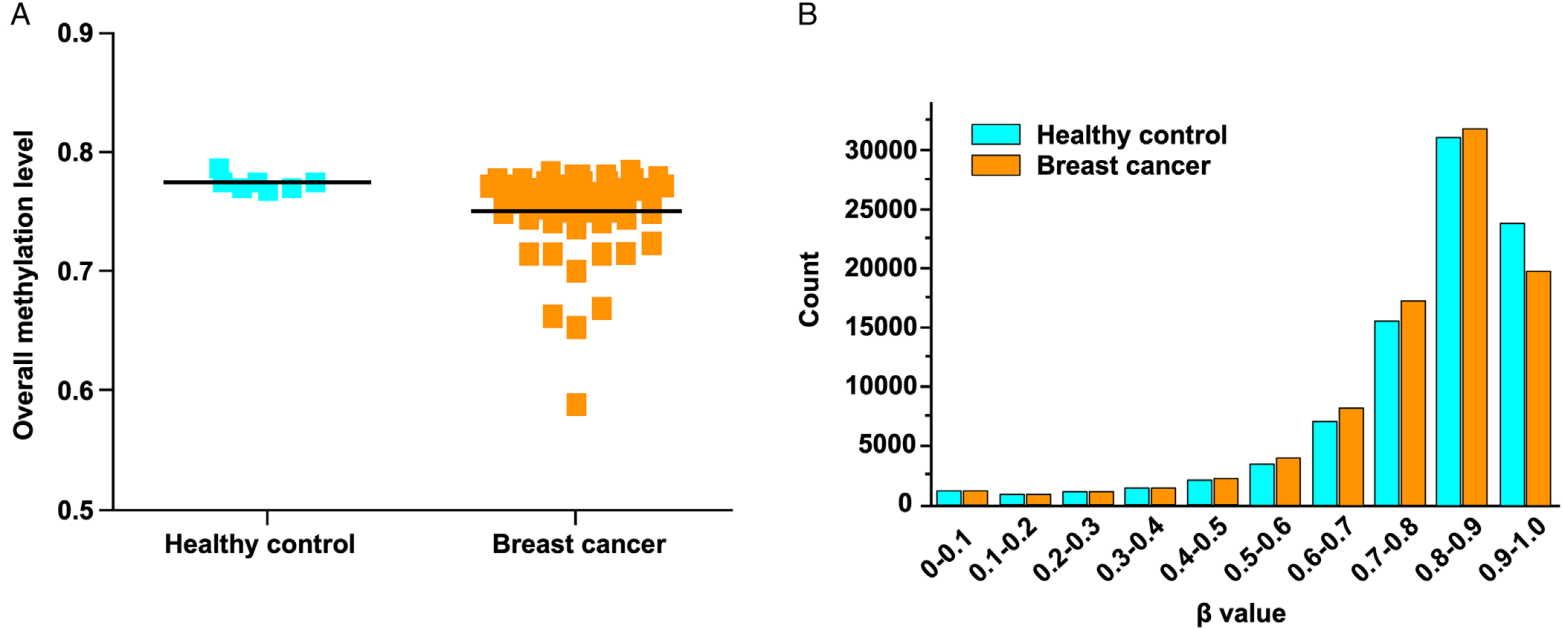
The vertical scatter plot and histogram show the whole‐genome DNA methylation level (A) and distribution (B) of circulating tumor DNA (ctDNA) from healthy controls and breast cancer patients

**FIGURE 3:**
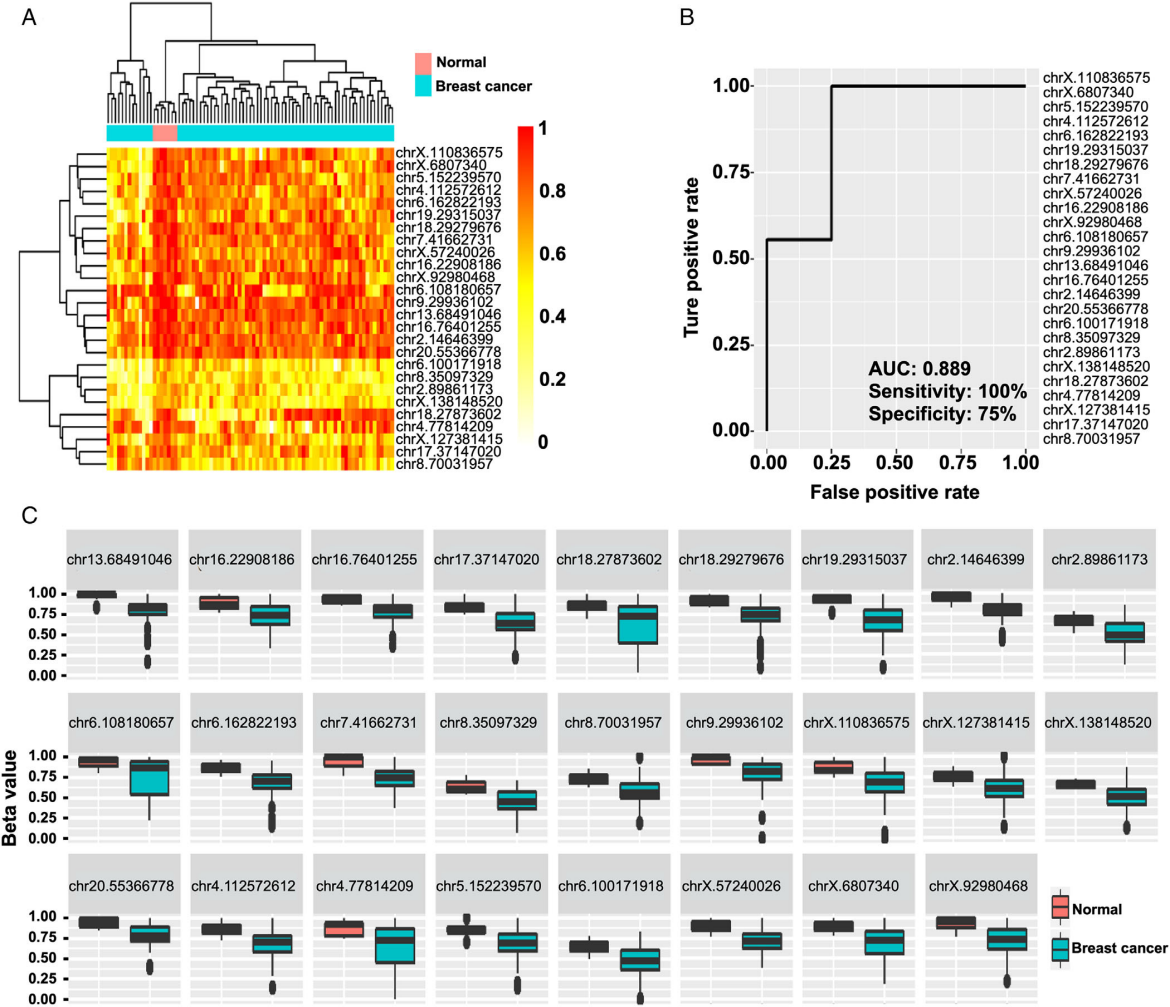
Methylation CpG sites selected for breast cancer diagnosis. (A) Heatmap of the DNA methylation levels of the methylated CpG sites in normal controls and breast cancer patients (mean difference >0.15, *p* < 0.05, standard deviation <0.1). (B) Receiver operating characteristic curve of a predictive model comprising 26 potential biomarkers. (C) The box plot shows the position of 26 candidate circulating tumor DNA (ctDNA) methylation CpG site biomarkers

To validate the efficacy of the method, we collected breast cancer samples and healthy controls (Table S1). We first analyzed the genome‐wide methylation patterns to compare the differences in the global methylation level and distribution of ctDNA from breast cancer patients and healthy controls. The results showed that the overall methylation level had no significant differences (Figure 2A). By dividing the methylation beta‐level (0–1) into 10 intervals, the results revealed that the breast cancer patients and healthy controls showed similar distribution patterns (Figure 2B). Then, we tried to compare the ctDNA methylation data of breast cancer patients with healthy controls to screen candidate CpG sites. Feature selection was performed by least absolute shrinkage and selection operator (LASSO)‐penalized logistic regression, resulting in 26 ctDNA methylation CpG sites as potential biomarkers for breast cancer diagnosis. The hierarchical clustering results suggested a precise classification of healthy individuals (*n* = 7) and patients (*n* = 74) in the training dataset using the methylation levels of these CpG sites (Figure 3A). The receiver operating characteristic (ROC) curve demonstrated that the sensitivity, specificity, and area under the curve (AUC) were 100%, 75%, and 0.889, respectively (Figure 3B). Meanwhile, the box plot shows the position of 26 potential biomarkers (Figure 3C).

### 2.3 ctDNA methylation biomarkers enabled the sensitive diagnosis of early‐stage breast cancer

**FIGURE 4:**
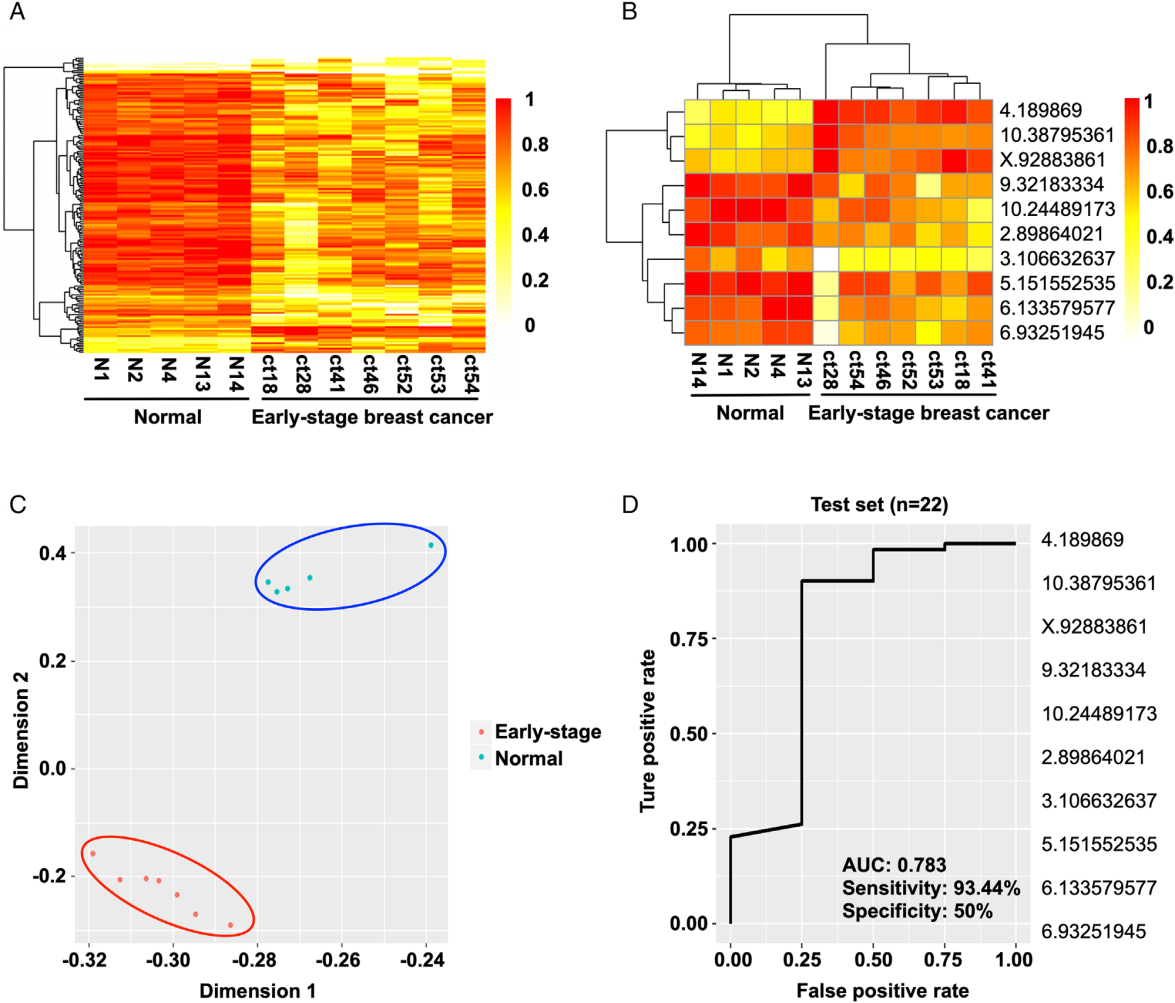
Methylation CpG sites in circulating tumor DNA (ctDNA) fragments selected for the early diagnosis of breast cancer. (A) Heatmap derived from cluster analysis of CpG sites (mean difference >0.15, *p* < 0.05, standard deviation <0.1). (B) Heatmap of the top 10 CpG sites between normal controls and early‐stage breast cancer samples. (C) Principal component analysis shows that these CpG sites can distinguish between normal controls and early‐stage breast cancer patients. (D) Receiver operating characteristic curve suggested the classification of normal controls and early‐stage breast cancer patients using the same 10 differentially methylated CpG sites that could be biomarkers

With these data in hand, we then decided to evaluate the early diagnostic ability of ctDNA methylation CpG sites. Specifically, we recruited seven early‐stage breast cancer patients from the Tianjin Medical University Cancer Institute and Hospital (TMUCIH), and the healthy control groups enrolled age‐matched Chinese women who were confirmed by physical examination. The heatmap results suggested a precise classification of healthy persons and early breast cancer patients (Figure 4A). Then, feature selection was performed by a random forest algorithm, resulting in 10 CpG sites as potential biomarkers for the early diagnosis of breast cancer (Figure 4B). The principal component analysis (PCA) data showed that healthy individuals and early‐stage breast cancer patients were precisely classified (Figure 4C). Furthermore, we validated the identified biomarkers for early diagnosis using independent cohorts named test set 2, which comprised 12 Chinese female patients with early‐stage breast cancer recruited from TMUCIH between May 2018 and October 2018. The ROC curve demonstrated that the AUC was 0.783, and the sensitivity and specificity were 93.44% and 50%, respectively (Figure 4D). Collectively, these results show that the 10 ctDNA methylation biomarkers obtained could sensitively distinguish early‐stage breast cancer.

### 2.4 ctDNA methylation biomarkers distinguished different subtypes of breast cancer

The heterogeneity of breast cancer suggests that the tumor may consist of phenotypically different cancer cell populations with diverse properties and expressions of various functional molecules. According to hormone and epidermal growth factor receptor expression status, breast cancer can be divided into several molecular subtypes: triple‐negative breast cancer (TNBC), luminal A (LA), luminal B (LB), human epidermal growth factor receptor 2 (HER2) enriched, normal‐like, and so forth.

**FIGURE 5:**
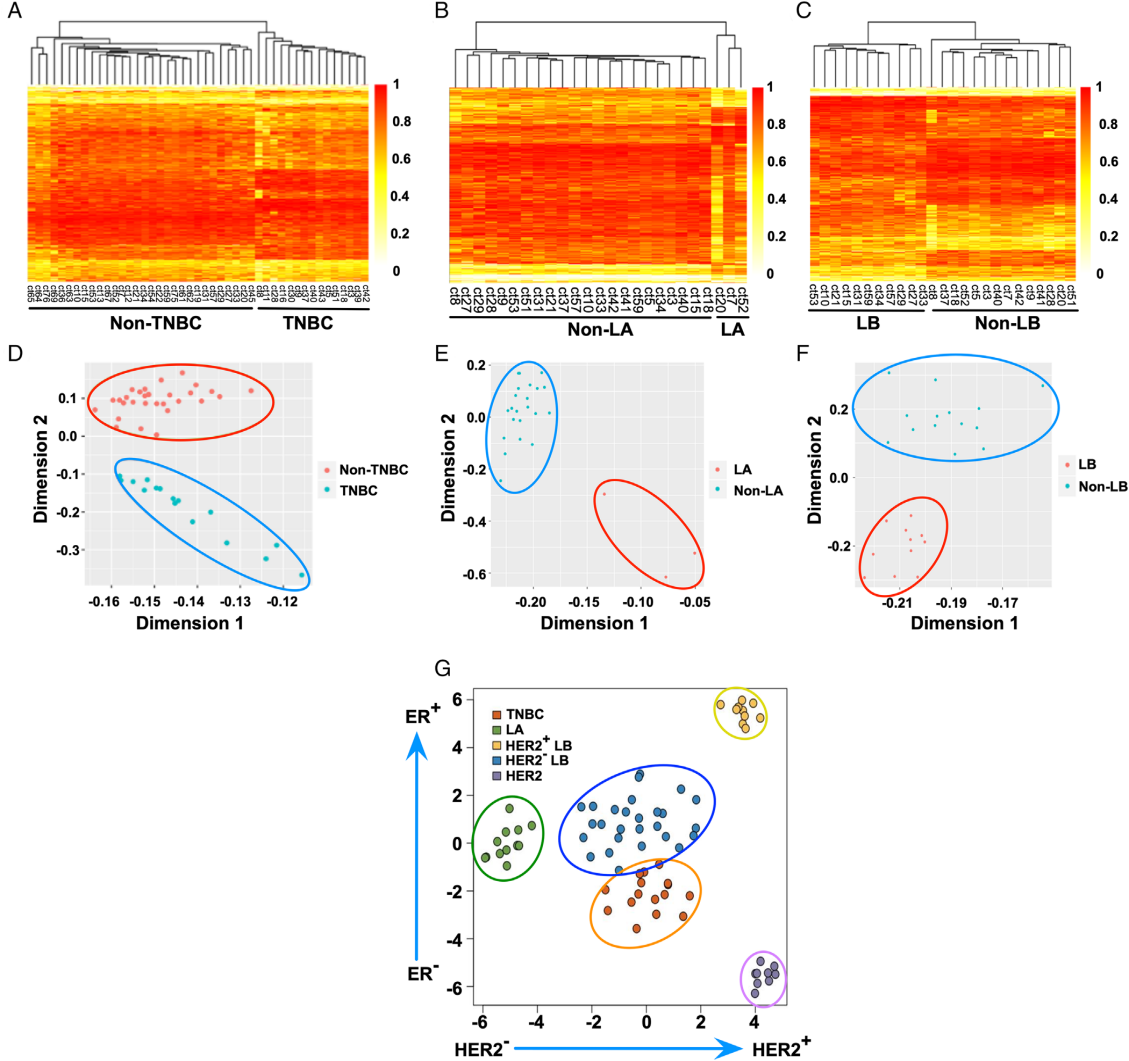
Methylation CpG sites in circulating tumor DNA (ctDNA) fragments selected for subtyping of breast cancer. (A) Heatmap of the DNA methylation levels of methylated CpG sites in triple‐negative breast cancer (TNBC) and non‐TNBC. (B) Heatmap of the DNA methylation levels of methylated sites in luminal A (LA) and non‐LA. (C) Heatmap of the DNA methylation levels of methylated CpG sites in luminal B (LB) and non‐LB. (D) Principal component analysis of TNBC and non‐TNBC. (E) Principal component analysis of LA and non‐LA. (F) Principal component analysis of LB and non‐LB. (G) *t*‐Distributed stochastic neighbor embedding plot suggested the classification of TNBC, LA, human epidermal growth factor receptor 2 (HER2)^+^ LB, HER2^–^ LB, and HER2 patients using independent The Cancer Genome Atlas dataset data

Encouraged by sensitive and specific early‐stage breast cancer detection, we constructed three breast cancer datasets to validate the subtyping ability of differentially methylated CpG sites, including TNBC patients and non‐TNBC controls, LA patients and non‐LA controls, and LB patients and non‐LB controls. For each dataset, we selected particular differentially methylated CpG sites for analysis. First, the heatmap plot showed that the obtained differentially methylated CpG sites could be used to divide the TNBC patients (*n* = 15) and non‐TNBC controls (*n* = 30) into two groups (Figure 5A). Then, the corresponding CpG sites performed well for subtyping LA patients (*n* = 3) and non‐LA controls (*n* = 22) (Figure 5B). Finally, the methylation level of CpG sites in ctDNA could also efficiently discriminate between LB patients (*n* = 11) and non‐LB controls (*n* = 14) (Figure 5C). Collectively, these results suggested that the differentially methylated CpG sites we identified could be used to subtype breast cancer by heatmap analysis.

Based on the encouraging outcomes, we performed PCA to further assess the capability of the as‐obtained ctDNA differentially methylated CpG sites to classify the subtypes of breast cancer. As shown in Figure 5D, the results indicated a precise classification of TNBC patients and non‐TNBC controls. In addition, two distinct clusters of LA patients versus non‐LA controls were separated (Figure 5E). Meanwhile, the PCA demonstrated that the LB patients were clustered together, and non‐LB controls were grouped at the top of the coordinate plot (Figure 5F).

To further validate our obtained biomarkers, we conducted external validation of the identified CpG sites using independent The Cancer Genome Atlas (TCGA) dataset. The *t*‐distributed stochastic neighbor embedding analysis indicated that TNBC (*n* = 15), LA (*n* = 12), HER2‐positive (HER2^+^) LB (*n* = 10), HER2‐negative (HER2^–^) LB (*n* = 26), and HER2 (*n* = 9) patients could be well distinguished (Figure 5G). Although further systematic evaluations are still needed, these preliminary results indicate that our study provides an unprecedented method to develop novel biomarkers complementary to traditional diagnostics for classifying various subtypes of breast cancer.

## 3 DISCUSSION

Breast cancer is the leading cause of death for women worldwide. X‐ray mammography has been used to screen breast cancer in many countries and has been evaluated as the best method thus far. However, the reports of overdiagnostics of the method are increasing.^29^ In addition, the American College of Radiology Breast Imaging Reporting and Data System (BI‐RADS) results provide negligible molecular information. Hence, it could not distinguish the precise details of the disease, for example, benign or malignant, early stage or other stages, TNBC or other subtypes, and so forth. More importantly, it is not very applicable in Asia because the structure of Asian women is quite different from women in Western countries. Therefore, it is critical to develop a noninvasive method for breast cancer diagnosis.

Among all noninvasive diagnostic methods, ctDNA methylation detection is the most promising method. Epigenetic abnormalities often lead to cancer, and many researchers have reported that 5‐methylcytosine is very important in detecting cancer.^30^ Furthermore, DNA methylation provides several more advantages than copy number variants and somatic mutation.^31^ ctDNA has many advantages and is widely studied.^32^ However, applying ctDNA in breast cancer early diagnosis remains highly challenging due to the contamination of background DNA from blood and low DNA methylation signals. To overcome the above obstacles, some researchers mixed several samples into one sample to obtain ctDNA levels to build the library.^33^ Unfortunately, those studies lost many messages and could not acquire individual differences.

We developed an improved ctDNA‐WGBS method to evenly profile whole‐genome methylation patterns from trace quantities of ctDNA in only 200 μl of plasma compared with the standard 5–20 ml of plasma: (i) Strect tubes were used to collect blood. The preservatives contained in Strect tubes stabilize nucleated blood cells, prevent the release of cellular genomic DNA, inhibit nuclease‐mediated degradation of ctDNA, and help improve the stability of ctDNA. (ii) A two‐step centrifugation was used: EDTA was added at the first centrifugation, and proteinase K was added at the second centrifugation. EDTA could inhibit the degradation of ctDNA by nucleases in blood, and proteinase K could improve ctDNA yield by releasing protein‐bound ctDNA. (iii) We compared the ctDNA separation method and found that the amount of ctDNA obtained was higher using the silica membrane‐based protocol than the conventional magnetic‐bead‐based protocol. (iv) During the extraction process, quality control needs to be performed to check whether the size of the enriched DNA fragments is appropriate. Only fragments of approximately 160–180 bp were applied in the subsequent ctDNA WGBS library construction. (v) To achieve less ctDNA requirement and reduce material loss, purified ctDNA was ligated with an adapter and converted using bisulfate with an “all‐in‐one tube” operation. That is, the process of end repair, dA‐tailing, adapter ligation, and bisulfite conversion was performed in the same tube. (vi) We increased the sequencing depths to obtain better signals for data analysis. With the above efforts, we could reduce background contamination and build a WGBS library from trace quantities of ctDNA.

Our analysis focused on detecting and classifying breast cancer from ctDNA to identify potential biomarkers at the whole‐genome scale. The results showed that ctDNA methylation is a potential biomarker for the early detection and molecular subtyping of breast cancer. Our work showed that different extraction methods could significantly influence the quality of ctDNA and are critical for identifying biomarkers with the ctDNA methylome. It is also very important to improve library construction to obtain enough peak‐enriched ctDNA for sequencing. In addition, increasing the sequencing depth is critical for signal amplification. With all these efforts, we could significantly improve the sensitivity and specificity of breast cancer prediction with the ctDNA methylome compared to recently reported work.^34^

According to industry research institutions, the cancer screening and early detection market is estimated to be more than 162 billion RMB yuan in China.^35^ We developed a noninvasive liquid biopsy technology with high breast cancer detection sensitivity while significantly reducing blood sample usage. The technology can significantly reduce the amount of blood used for testing, and the sample consumption is only one‐tenth of that of commercial kits, which can improve patient compliance. Moreover, the noninvasive liquid biopsy strategy combined with conventional biomarkers might reduce the frequency of unnecessary biopsies in the clinic. The protocol was effective at developing novel sensitive and specific biomarkers for the diagnosis, early detection, and molecular subtyping of breast cancer. We anticipate that the improved ctDNA‐WGBS method and diagnostic biomarkers of breast cancer have substantial clinical translation potential.

Several limitations also need to be acknowledged in this study. First, the blood samples from breast cancer patients and healthy controls were relatively small. Furthermore, the lack of multicenter patient cohorts for validation during the screening of breast cancer ctDNA methylation biomarkers may lead to some potential bias. We will focus on investigating the optimal ctDNA methylation biomarkers with large‐scale ctDNA samples from multicenter patient cohorts in future work.

## 4 MATERIALS AND METHODS

### 4.1 Sample collection

We recruited two cohorts of Chinese breast cancer patients from the Tianjin Medical University Cancer Institute and Hospital (TMUCIH): (i) Test set 1 consisted of early‐stage (*n* = 7) and advanced‐stage (*n* = 44) breast cancer female patients of Chinese descent from the TMUCIH between December 2016 and December 2017. In sum, we enrolled 51 Chinese female patients with early/advanced breast cancer (50.87 ± 11.05 as the mean age ± standard deviation [SD]) in test set 1. (ii) Test set 2 comprised Chinese female patients with early‐stage (*n* = 12) and advanced‐stage (*n* = 11) breast cancer recruited from the TMUCIH between May 2018 and October 2018. For test set 2, 23 Chinese female patients with early/advanced breast cancer (53.47 ± 8.88 as the mean age ± SD) were recruited. Collectively, there were 74 female patients with breast cancer enrolled in this study.

The healthy control groups enrolled age‐matched Chinese women without cancer from the Beijing Institute of Genomics (BIG), confirmed by physical examination, ultrasound scans of breast, and mammographic screening. Informed consent or a waiver of consent was obtained from each participant. In total, seven healthy women were recruited into healthy control groups.

### 4.2 Phenotypic evaluation

The diagnosis of breast cancer was histologically confirmed. We conducted phenotypic analyses of age, menopausal status, stage, pathology features, and molecular subtype in the two cohorts. Information on estrogen receptor (ER) and progesterone receptor (PR) status was assessed by immunohistochemistry (IHC) and extracted from pathology reports. Specifically, the paraffin samples were cut into 4‐μ e paraffin samples were cut into hology features, and molecular subtype in the two cohorts. Information on estrogen receptor (E mol/L citrate buffer (pH = 6.0) for 10 min for antigen retrieval. After cooling, the slides were washed with PBS (pH = 7.4) and incubated with 0.3% H_2_O_2_ solution for 10 min to block endogenous peroxidase activity. Primary polyclonal antibodies against ER, PR, and HER2 were applied to the samples and incubated overnight at 4al r subtype in the two cohorts. Informath PBS, and the antibody reaction was visualized using a fresh substrate solution containing diaminobenzidine. Images were captured by a Leica DC500 camera (Solms, Germany) on a microscope equipped with Leica DMRA2 fluorescent optics under 10 orts. Innification, and the expression level was defined as the mean density of ER‐, PR‐, and HER2‐specific staining/area. Tumors were classified as ER/PR‐negative if IHC staining of tumor cell nuclei was less than 1% reactivity. HER2 was assessed through IHC or fluorescence in situ hybridization (FISH) if the IHC results were borderline. The clinical grouping of molecular subtypes was defined by the status of hormone receptor and HER2 according to the St. Gallen 2017 criteria.^36^

### 4.3 Circulating tumor DNA extraction

Whole blood of healthy and cancer samples was collected using ctDNA blood collection tubes (Strect, USA). Plasma was then separated by centrifugation at 1900 × *g* for 10 min and 18,000× *g* for 10 min at room temperature with EDTA and protinease K. ctDNA was extracted from plasma using the QIAamp Circulating Nucleic Acid Kit (55114) or magnetic beads method according to the manufacturer's instructions. The ctDNA concentration was determined using a Qubit dsDNA HS Assay Kit (Life Technologies, Q32851). The extracted ctDNA was stored at −80°C for further library construction.

### 4.4 ctDNA methylation library construction

As shown in Figure 1D, we used the DNA to build the methylation library. We mixed 20 ng ctDNA and 1/1000–5/1000 ng unmethylated lambda DNA (D1521, Promega). Unmethylated lambda DNA was sheared by a Covaris S220 instrument (Life Technologies, Thermo Fisher Scientific), and the size of fragments was approximately 200 bp. First, DNA fragments were synthesized via end‐repair and A‐tailing reactions. Second, NEBNext Ultra Ligation Module (E7445S/L, NEB) reagent was used to ligate the methylation adaptor. Then, we used the EZ DNA Methylation‐Gold Kit (D5005, Zymo) to bisulfite‐converted DNA according to the manufacturer's recommendations. Next, DNA was amplified by PCR, which was carried out using primers. The primers came from Illumina next‐generation sequencing. PCR was performed to amplify the fragments. DNA gel extraction and magnetic bead extraction were performed to compare the different influences of library quality. Finally, we selected effective captures (∼290 bp) as the resulting library using Agencourt AMPure XP beads (Beckman Coulter). All binding and washing procedures were performed at room temperature. The purity of the libraries was analyzed by Qubit dsDNA HS assay (Thermo Fisher). A total of 100 G data were obtained for each sample.

### 4.5 Quality control and mapping of WGBS data

The raw sequencing reads (fastq format) were trimmed to remove sequencing adapters, amplification primers, and low‐quality bases in read ends using trim_galore (version 0.4.2) (http://www.bioinformatics.babraham.ac.uk/projects/trim_galore). After quality control, the reads were mapped to the human reference genome (hg19) using Bismark (version 0.14.2) with default parameters.^37^ Next, the PCR duplicates were also removed using Bismark (version 0.14.2) with default parameters.^37^ The bisulfite conversion rate was calculated by the spike‐in of totally unmethylated lambda DNA. Only the mapped and duplicate‐removed reads (bam format) were used for the subsequent bioinformatics analysis.

### 4.6 Data processing and analysis

We sequenced DNA methylation libraries by using X‐ten systems (Illumina). The total reads were assessed by FASTQC (version 0.11.8). The raw sequencing reads were then cleaned by Trim Galore (version 0.4.5) to clip sequencing adapters and low‐quality reads. Subsequently, the trimmed reads were aligned against the human reference genome using the Bismark (version 0.14.3) tool. Finally, differential methylation analysis of the DNA methylation sequencing data was performed by the limma package. For each dataset, the top CpG sites with *p*‐value <0.05 and absolute mean methylation difference >0.15 were selected. Sites were annotated by PeakAnnotator (version 1.4).

## CONFLICT OF INTEREST

The authors declare no competing interests.

## ETHIC STATEMENT

Our study was approved by the Ethics Committee of Tianjin Medical University Cancer Institute and Hospital (approved protocol no. 20210205). All individuals were adequately informed and signed an informed consent form before participating in the study.

## AUTHOR CONTRIBUTIONS

Luo Hai participated in data analysis, original draft writing, and review & editing. Ling yu Li and Zongzhi Liu participated in sample collection, data analysis, and paper writing. Zhongsheng Tong and Yingli Sun designed the experiment and revised the article. All authors read and approved the final manuscript.

## Supporting information

Supporting Information

## Data Availability

The code and data that support the findings of this study are openly available in GitHub at https://github.com/zhq921/cfWGBS-bioinfo-pip. The raw sequencing data reported in this manuscript are publicly available at the Genome Sequence Archive under accession number CRA001142 (https://ngdc.cncb.ac.cn/gsa/browse/CRA001142). All the data and materials are available from the corresponding author upon reasonable request.
