# Supplementary material for: Whole‐genome circulating tumor DNA methylation landscape reveals sensitive biomarkers of breast cancer
Source: MedComm (2020). 2022 Jun 17;3(3):e134. doi: 10.1002/mco2.134 (PMC9205580; doi:10.1002/mco2.134)
Supplement: Supplementary file 1 — Supporting Information [file MCO2-3-e134-s001.docx]

Supporting Information

of

**Whole-genome circulating tumor DNA methylation landscape reveals sensitive biomarkers of breast cancer**

Luo Hai^#^, Lingyu Li^#^, Zongzhi Liu^#^, Zhongsheng Tong^*^, Yingli Sun^*^

^1^ Central Laboratory, National Cancer Center/National Clinical Research Center for Cancer/Cancer Hospital & Shenzhen Hospital, Chinese Academic of Medical Sciences and Peking Union Medical College, Shenzhen, China

^2^ Department of Breast Oncology, Tianjin Medical University Cancer Institute and Hospital, Tianjin, China

^3^ University of Chinese Academy of Sciences, Beijing, China

^4^ CAS Key Laboratory of Genome Sciences and Information, Beijing Institute of Genomics, Chinese Academy of Sciences, Beijing, China

**Correspondence**

Yingli Sun, Central Laboratory, National Cancer Center/National Clinical Research Center for Cancer/Cancer Hospital & Shenzhen Hospital, Chinese Academic of Medical Sciences and Peking Union Medical College, Shenzhen 518116, China

Email: scaroll99@hotmail.com

Zhongsheng Tong, Department of Breast Oncology, Tianjin Medical University Cancer Institute and Hospital, Tianjin 300060, China

Email: tongzhongsheng@tjmuch.com

^#^ These authors contributed equally to this work.

**TABLE S1** **The clinic information and raw data information of the breast cancer patients and healthy controls.**

| **Group** | **Serial ID** | **Sample ID** | **Staging** | **Sample source** | **Molecular subtype** | **Cs methylated in CpG context** | **Mean coverage** | **Conversion rate** |
| --- | --- | --- | --- | --- | --- | --- | --- | --- |
| **Test set 1** | 1 | ct18 | Early | Plasma | TNBC | 79.80% | 2.6 | 99.78% |
|  | 2 | ct28 | Early | Plasma | TNBC | 81.80% | 3.4 | 99.29% |
|  | 3 | ct41 | Early | Plasma | - | 80.50% | 2.0 | 99.08% |
|  | 4 | ct46 | Early | Plasma | - | 81.90% | 2.4 | 99.00% |
|  | 5 | ct52 | Early | Plasma | LA | 67.90% | 2.1 | 99.12% |
|  | 6 | ct53 | Early | Plasma | LB | 80.60% | 2.7 | 98.63% |
|  | 7 | ct54 | Early | Plasma | - | 80.90% | 3.3 | 98.92% |
|  | 8 | ct10 | Advanced | Plasma | LB | 81.00% | 3.0 | 99.46% |
|  | 9 | ct11 | Advanced | Plasma | TNBC | 81.60% | 3.1 | 99.34% |
|  | 10 | ct12 | Advanced | Plasma | - | 82.30% | 2.9 | 99.28% |
|  | 11 | ct15 | Advanced | Plasma | LB | 82.10% | 3.2 | 99.29% |
|  | 12 | ct17 | Advanced | Plasma | - | 82.90% | 3.2 | 98.49% |
|  | 13 | ct19 | Advanced | Plasma | - | 79.90% | 2.5 | 99.80% |
|  | 14 | ct20 | Advanced | Plasma | LA | 82.10% | 3.2 | 99.36% |
|  | 15 | ct25 | Advanced | Plasma | - | 81.00% | 3.5 | 99.28% |
|  | 16 | ct27 | Advanced | Plasma | LB | 82.50% | 3.1 | 99.16% |
|  | 17 | ct29 | Advanced | Plasma | LB | 79.10% | 2.4 | 99.83% |
|  | 18 | ct3 | Advanced | Plasma | TNBC | 80.40% | 2.4 | 99.85% |
|  | 19 | ct30 | Advanced | Plasma | TNBC | 81.50% | 2.9 | 98.56% |
|  | 20 | ct31 | Advanced | Plasma | LB | 82.00% | 3.0 | 98.62% |
|  | 21 | ct33 | Advanced | Plasma | LB | 81.80% | 3.0 | 98.78% |
|  | 22 | ct34 | Advanced | Plasma | LB | 81.20% | 2.2 | 99.09% |
|  | 23 | ct36 | Advanced | Plasma | - | 81.90% | 2.2 | 98.79% |
|  | 24 | ct37 | Advanced | Plasma | TNBC | 79.90% | 2.1 | 99.06% |
|  | 25 | ct39 | Advanced | Plasma | TNBC | 79.90% | 2.0 | 99.11% |
|  | 26 | ct40 | Advanced | Plasma | TNBC | 80.40% | 2.2 | 98.91% |
|  | 27 | ct42 | Advanced | Plasma | TNBC | 79.10% | 1.5 | 98.44% |
|  | 28 | ct43 | Advanced | Plasma | TNBC | 80.90% | 2.3 | 98.64% |
|  | 29 | ct45 | Advanced | Plasma | - | 81.00% | 1.7 | 98.52% |
|  | 30 | ct5 | Advanced | Plasma | TNBC | 81.70% | 2.8 | 98.73% |
|  | 31 | ct51 | Advanced | Plasma | TNBC | 79.10% | 2.2 | 99.05% |
|  | 32 | ct57 | Advanced | Plasma | LB | 80.40% | 2.5 | 98.99% |
|  | 33 | ct59 | Advanced | Plasma | LB | 82.20% | 2.5 | 98.82% |
|  | 34 | ct63 | Advanced | Plasma | - | 79.60% | 2.8 | 98.86% |
|  | 35 | ct64 | Advanced | Plasma | - | 79.00% | 1.8 | 99.22% |
|  | 36 | ct65 | Advanced | Plasma | - | 80.20% | 2.9 | 98.76% |
|  | 37 | ct69 | Advanced | Plasma | - | 82.80% | 2.2 | 98.71% |
|  | 38 | ct7 | Advanced | Plasma | LA | 67.60% | 1.4 | 98.88% |
|  | 39 | ct75 | Advanced | Plasma | - | 79.60% | 2.0 | 99.40% |
|  | 40 | ct76 | Advanced | Plasma | - | 66.10% | 1.8 | 99.02% |
|  | 41 | ct8 | Advanced | Plasma | TNBC | 78.40% | 1.8 | 98.72% |
|  | 42 | ct9 | Advanced | Plasma | TNBC | 80.30% | 2.0 | 98.80% |
|  | 43 | ct78 | Advanced | Plasma | - | 80.00% | 2.7 | 99.21% |
|  | 44 | ct79 | Advanced | Plasma | - | 81.60% | 2.3 | 98.97% |
|  | 45 | ct81 | Advanced | Plasma | - | 80.60% | 2.7 | 98.45% |
|  | 46 | ct82 | Advanced | Plasma | - | 80.30% | 2.2 | 98.78% |
|  | 47 | ct83 | Advanced | Plasma | - | 80.20% | 2.2 | 99.28% |
|  | 48 | ct85 | Advanced | Plasma | - | 81.30% | 1.7 | 98.78% |
|  | 49 | ct86 | Advanced | Plasma | - | 58.80% | 2.5 | 99.08% |
|  | 50 | ct87 | Advanced | Plasma | - | 81.10% | 2.2 | 98.27% |
|  | 51 | ct88 | Advanced | Plasma | - | 79.90% | 2.5 | 98.83% |
| **Test set 2** | 52 | ct13 | Early | Plasma | - | 82.00% | 3.1 | 98.59% |
|  | 53 | ct16 | Early | Plasma | TNBC | 81.80% | 2.9 | 99.07% |
|  | 54 | ct21 | Early | Plasma | LB | 80.10% | 2.2 | 99.09% |
|  | 55 | ct22 | Early | Plasma | - | 82.70% | 2.8 | 98.05% |
|  | 56 | ct61 | Early | Plasma | - | 82.90% | 3.1 | 98.76% |
|  | 57 | ct62 | Early | Plasma | - | 81.40% | 2.7 | 99.16% |
|  | 58 | ct66 | Early | Plasma | - | 81.00% | 3.1 | 98.76% |
|  | 59 | ct67 | Early | Plasma | - | 69.60% | 3.0 | 98.78% |
|  | 60 | ct68 | Early | Plasma | - | 80.80% | 2.2 | 99.32% |
|  | 61 | ct92 | Early | Plasma | - | 81.50% | 2.2 | 98.28% |
|  | 62 | ct101 | Early | Plasma | - | 80.40% | 2.6 | 99.18% |
|  | 63 | ct108 | Early | Plasma | - | 77.80% | 2.6 | 98.76% |
|  | 64 | ct89 | Advanced | Plasma | - | 81.50% | 2.7 | 99.24% |
|  | 65 | ct94 | Advanced | Plasma | - | 80.40% | 2.3 | 99.19% |
|  | 66 | ct95 | Advanced | Plasma | - | 79.80% | 2.4 | 98.74% |
|  | 67 | ct97 | Advanced | Plasma | - | 81.30% | 2.5 | 98.54% |
|  | 68 | ct98 | Advanced | Plasma | - | 81.80% | 2.7 | 98.89% |
|  | 69 | ct100 | Advanced | Plasma | - | 78.50% | 2.8 | 99.15% |
|  | 70 | ct104 | Advanced | Plasma | - | 81.40% | 3.1 | 98.77% |
|  | 71 | ct105 | Advanced | Plasma | - | 81.80% | 3.1 | 98.65% |
|  | 72 | ct107 | Advanced | Plasma | - | 79.40% | 2.8 | 99.21% |
|  | 73 | ct110 | Advanced | Plasma | - | 82.50% | 2.6 | 97.85% |
|  | 74 | ct112 | Advanced | Plasma | - | 76.30% | 2.6 | 99.36% |
| Control group | 75 | N1 | Normal | Plasma | N.A. | 78.90% | 2.2 | 99.03% |
|  | 76 | N2 | Normal | Plasma | N.A. | 80.70% | 2.2 | 99.08% |
|  | 77 | N4 | Normal | Plasma | N.A. | 81.70% | 3.5 | 98.93% |
|  | 78 | N6 | Normal | Plasma | N.A. | 71.90% | 2.7 | 99.18% |
|  | 79 | N11 | Normal | Plasma | N.A. | 79.50% | 1.9 | 98.80% |
|  | 80 | N13 | Normal | Plasma | N.A. | 79.20% | 2.0 | 99.11% |
|  | 81 | N14 | Normal | Plasma | N.A. | 80.80% | 3.0 | 98.84% |
